# The application of visceral adiposity index in identifying type 2 diabetes risks based on a prospective cohort in China

**DOI:** 10.1186/1476-511X-13-108

**Published:** 2014-07-08

**Authors:** Chen Chen, Yan Xu, Zhi-rong Guo, Jie Yang, Ming Wu, Xiao-shu Hu

**Affiliations:** 1 School of Public Health, Southeast University, Nanjing, Jiangsu 210009, China; 2 Department of Chronic Diseases Control, Jiangsu Provincial Center for Disease Control and Prevention, Nanjing 210009, China; 3 Department of Immune Planning, Jiangsu Provincial Center for Disease Control and Prevention, Nanjing 210009, China; 4 School of Public Health, Soochow University, Suzhou, Jiangsu 215123, China; 5 Jiangsu Food and Drug Administration, Nanjing, Jiangsu 210009, China; 6 Jiangsu Provincial Center for Disease Control and Prevention, No. 172, Jiangsu Road, Nanjing 210009, China

**Keywords:** Diabetes, Visceral adiposity index, Body mass index, Waist circumference, Waist-to-height ratio

## Abstract

**Background:**

Visceral adiposity index (VAI), a novel sex-specific index for visceral fat measurement, has been proposed recently. We evaluate the efficacy of VAI in identifying diabetes risk in Chinese people, and compare the predictive ability between VAI and other body fatness indices, i.e., waist circumference (WC), body mass index (BMI) and waist- to- height ratio (WHtR).

**Methods:**

Participants (n = 3,461) were recruited from an ongoing cohort study in Jiangsu Province, China. Hazard ratio (HR) and corresponding 95% confidence interval (CI) between diabetes risk and different body fatness indices were evaluated by Cox proportional hazard regression model. Receiver operating characteristic (ROC) curve and area under curve (AUC) were applied to compare the ability of identifying diabetes risk between VAI, WC, WHtR and BMI.

**Results:**

A total number of 160 new diabetic cases occurred during the follow-up, with an incidence of 4.6%. Significant positive associations were observed for VAI with blood pressure, fasting plasma glucose, triglyceride, WC, BMI and WHtR. Moreover, increased VAI was observed to be associated with higher diabetes risk with a positive dose–response trend (*p* for trend < 0.001). As compared to individuals with the lowest VAI, those who had the highest VAI were at 2.55-fold risk of diabetes (95% CI: 1.58-4.11). The largest AUC was observed for VAI, following by WC, WHtR and BMI.

**Conclusions:**

VAI is positively associated with the risk of diabetes. Compared to other indices for body fatness measurements, VAI is a better and convenience surrogate marker for visceral adipose measurement and could be used in identifying the risk of diabetes in large-scale epidemiologic studies.

## Introduction

Diabetes has become a worldwide public health problem seriously threatening human health [1]. There was a rapid rising trend in the prevalence of diabetes in China during the past decades [2]. The results of 2007–2008 National Diabetic Epidemiological Survey showed that the prevalence of diabetes in China was 9.7%, and estimated that the number of Chinese diabetic patients is 92.4 million, which indicates that China is not only a region with the fastest growing speed of diabetes, but also is the country with the largest diabetes population [3].

Many studies have confirmed that obesity plays an important role in the development of diabetes. Excess fat may lead to insulin resistance (IR) and abnormal glucose metabolism [4,5]. Oti et al. found that obesity is closely related to high blood glucose particularly among African women [6]. Matsuda summarized that adipose tissue is a main source of reactive oxygen species, which may contribute to the development of obesity-associated IR and cause type 2 diabetes as a consequence [7]. However, obese people may vary in their body fat distribution and disease risk, and regional adipose tissue distribution was found as a key role in explaining the relationship between adiposity and glycometabolism. It is reported that individuals with high visceral adiposity are associated with increased risks of IR and metabolic disorders, and are more likely to suffer from diabetes [8,9]. Bu et al. found that average IR in visceral obesity group was significant higher than that in non-obese group [10].

In most studies, body fat was measured by waist circumference (WC), body mass index (BMI) and waist-to-height ratio (WHtR), but these indices are not able to distinguish between subcutaneous fat and visceral fat [11]_._ For instance, even with BMI in normal range, individuals may still have high level of visceral adipose tissue (VAT). For measuring the volume of visceral fat, magnetic resonance imaging (MRI) and computed tomography (CT) has been recognized as the gold standard for quantitative detection [12], however, these two methods are inconvenience and expensive, and are obviously not suitable for large-scale epidemiological studies. Recently, Amato et al. proposed a novel sex-specific index for visceral fat measurement, which is calculated on the basis of WC, BMI, triglyceride (TG) and high density lipoprotein cholesterol (HDL-C), and is termed as visceral adiposity index (VAI). They observed that VAI was highly correlated with body visceral adiposity measured by MRI [13]. VAI combined both physical and metabolic parameters to evaluate body fatness, which may reflect visceral adiposity better than other indices. Bozorgmanesh et al. found that VAI has good prediction effect on diabetes in Tehran people [14]. Although VAI has been suggested as a useful and convince surrogate marker for visceral adiposity, it’s efficacy in predicting diabetes risk still needs to be further confirmed, moreover, no previous studies have been conducted among Chinese population. In this study, we took advantage of an ongoing prospective study in Jiangsu Province, China; it aims to further evaluate the efficacy of using VAI to identify diabetes risk in Chinese people, and to compare the risk predictive ability between VAI and other body fatness indices, i.e., WC, BMI and WHtR.

## Materials and methods

### Study population

The study of Prevention of Multiple Metabolic Disorders and Metabolic Syndrome in Jiangsu Province is an ongoing prospective cohort study aimed to estimate the prevalence of metabolic syndrome (MS) and the incidence of type 2 diabetes in Jiangsu Province, China. The study design has been previously described in detail [15-18]. Briefly, this cohort has been established since early 2000s in Jiangsu. A total number of 6,400 participants aged 35–74 years was randomly selected for the baseline survey by a multi-stage sampling method, and 92% of them (n = 5,888) completed the first round survey.

During 2006–2009, subjects (n = 4,582) who had already been followed up for more than 5 years were invited for the second round survey and 3,847 subjects participated. As the primary endpoint of this study was the occurrence of diabetes, we excluded subjects who have been diagnosed with diabetes at baseline. In total, 3,461 subjects completed the follow-up and were involved in the present analysis. The study was approved by the ethical committee of Jiangsu Provincial Center for Disease Control and Prevention.

### Data collection

With written informed consent, a pre-tested questionnaire was used to collect epidemiologic information by trained interviewers. The information obtained including demographic information, lifestyle risk factors, personal medical history and family history of diabetes (DM-FH).

Anthropometric measurements were conducted at the time of interview including weight, height, WC and blood pressure. Body weight and height were measured by standard methods, and recorded to the nearest 0.1 kg and 0.1 cm, respectively. BMI was calculated as the weight in kilograms divided by the square of the height in meters. WC was measured two times at 1 cm above the umbilicus level at minimal respiration. WHtR was calculated as dividing WC by height. Systolic blood pressure (SBP) and diastolic blood pressure (DBP) were examined 3 times at 30-second intervals by trained doctors, using a standardized mercury sphygmomanometer on the right arm after resting for 5 minutes in a sitting position [19].

Blood samples were collected in the morning after at least 8 hours of overnight fasting. All plasma and serum samples were frozen at -80°C until laboratory test was performed. Laboratory tests were conducted in qualified labs in Jiangsu Provincial Center for Disease Control and Prevention, including fasting plasma glucose (FPG), TG, HDL-C. FPG was measured by an enzymatic colorimetric method with glucose oxidize. HDL-C and TG were assessed enzymatically using an automatic biochemistry analyzer (Hitachi Inc, Tokyo, Japan) and commercial reagents [18].

### Diabetes diagnostic criteria

All newly occurred diabetes patients during the follow-up period were diagnosed according to the criteria of WHO, 1999 [20]: FPG no less than 7 mmol/L (or 126 mg/dL), or have been diagnosed as diabetes by a county level hospital or above.

### Statistical analysis

Data were entered into the computer double parallel using Epidata 3.0, cleaned and analyzed using SPSS 17.0. The start of follow-up was defined as the date of first round survey; for newly occurred diabetic patients, follow-up ended at the time of diabetes occurrence. Mean and standard deviation (SD) were calculated for continuous variables. Analysis of variance was used for comparison between groups if they were in normal distribution. For variables in skewed distribution, we calculated their medians and inter-quartile ranges, and used rank sum test for the comparison between groups. Categorical variables were calculated for percentages and chi-square test was used for statistical analysis.

Confounders were selected based on previous knowledge on diabetes risk factors and our preliminary analysis, including age, gender, smoking, alcohol drinking, SBP, DBP and DM-FH.

According to Amato’s research [13], VAI was defined as:

$$\begin{array}{ll} \mathrm{Male}:\mathrm{VAI}= & \left(\frac{\mathrm{WC}}{39.68+\left(1.88\times \mathrm{BMI}\right)}\right)\times \left(\frac{\mathrm{TG}}{1.03}\right)\\ & \times \left(\frac{1.31}{\mathrm{HDL}‒C}\right) \end{array}$$

$$\begin{array}{ll} \mathrm{Female}:\mathrm{VAI}= & \left(\frac{\mathrm{WC}}{36.58+\left(1.89\times \mathrm{BMI}\right)}\right)\times \left(\frac{\mathrm{TG}}{0.81}\right)\\ & \times \left(\frac{1.52}{\mathrm{HDL}‒C}\right) \end{array}$$

VAI = 1 stands for subjects are healthy, non-obese, with normal adipose distribution and normal TG and HDL-C levels.

In the present analysis, VAI was grouped by quartiles (the cut-off point was selected from general population without diabetes). According to Cooperative Meta-analysis Group of China Obesity Task Force, BMI were divided into three groups, normal (<24 kg/m^2^), overweight (24–27.9 kg/m^2^) and obesity (≥28 kg/m^2^) [21]. Cut-off point of WC for men and women were 85 cm and 80 cm, respectively [21]. WHtR less than 0.5 was considered as normal [22]. Hazard ratio (HR) and corresponding 95% confidence intervals (CIs) were calculated by Cox proportional hazard regression model to evaluate the associations between different body fatness indices and diabetes risk. Dummy variables were used to calculate HR for each categorical variable. The trend test was performed by assigning scores to different categorical variables and treated the categorical variables as continuous variables in the Cox regression model. Receiver operating characteristic (ROC) curve was applied to compare the predictive effect between VAI, WC, WHtR and BMI for diabetes risk. The area under curve (AUC) was calculated for comparison between four body fatness indices. Significant level for statistics analysis was set as *p* < 0.05.

## Results

In total, 1,406 males and 2,055 females were involved in the present analysis. The median follow-up time was 5.8 years. Table 1 shows the baseline epidemiologic characteristics of 3,461 participants by gender. The average age of men and women was 50.60 and 49.95 years old, respectively. Men's smoking and drinking rate was higher than that of women. No significant difference was observed in SBP, DBP and FPG between males and females. But for VAI, we found women were more likely to have higher VAI than men. During the follow-up, a total number of 160 newly occurred diabetes cases were identified during the follow-up period (4.6%), 60 of them were males and 100 were females.

**Table 1:** The epidemiologic characteristic of study participants by gender

**Variables**	**Male (%)**	**Female (%)**	**Total (%)**
	**(N = 1,406)**	**(N = 2,055)**	**(N = 3,461)**
**Age** (years)			
Mean ± SD	50.60 ± 9.80	49.95 ± 10.07	50.21 ± 9.97
< 45	421 (29.9%)	700 (34.1%)	1121 (32.4%)
45 ~ 54	513 (36.5%)	706 (34.4%)	1219 (35.2%)
≥55	472 (33.6%)	649 (31.6%)	1121 (32.4%)
**SBP** (mmHg)			
Mean ± SD	126.61 ± 19.57	124.84 ± 19.92	125.56 ± 19.79
**DBP** (mmHg)			
Mean ± SD	81.39 ± 11.26	78.63 ± 10.46	79.75 ± 10.87
**FPG** (mmol/L)			
Mean ± SD	5.11 ± 0.63	5.17 ± 0.64	5.15 ± 0.63
**Alcohol drinking**			
No-drinking	745 (53.0%)	1960 (95.4%)	2705 (78.2%)
Ex-drinking	26 (1.8%)	0 (0%)	26 (0.8%)
Drinking	635 (45.2%)	95 (4.6%)	730 (21.1%)
**Smoking history**			
No-smoking	583 (41.5%)	1918 (93.3%)	2501 (72.3%)
Ex-smoking	155 (11.0%)	11 (0.5%)	166 (4.8%)
Smoking	668 (47.5%)	126 (6.1%)	794 (22.9%)
**DM-FH (%)**	66 (4.7%)	114 (5.5%)	180 (5.2%)
**VAI**			
I (≤1.047)	528 (37.6%)	320 (15.6%)	848 (24.5%)
II (1.048-)	371 (26.4%)	484 (23.6%)	855 (24.7%)
III (1.643-)	265 (18.8%)	606 (29.5%)	871 (25.2%)
IV (≥2.66)	242 (17.2%)	645 (31.4%)	887 (25.6%)
**Diabetes**	60 (4.3%)	100 (4.9%)	160 (4.6%)

SD, standard deviation; SBP, systolic blood pressure; DBP, diastolic blood pressure; FPG, fasting plasma glucose; DM-FH, family history of diabetes; VAI, visceral adipose index.

Table 2 presents the associations between VAI level and different metabolic variables. Significant positive dose–response relationship was observed for VAI with SBP, DBP, FPG, TG, WC, BMI and WHtR (*p for trend* < 0.001). On the contrary, HDL-C was observed negatively correlated with VAI level (*p* < 0.001).

**Table 2:** Characteristics of metabolic variables in different VAI groups

	**VAI**				***p *** **for trend**
	**I (≤1.047)**	**II (1.048-)**	**III (1.643-)**	**IV (≥2.66)**	
**SBP (mmHg)**					
Male	125.08 ± 19.95	125.43 ± 19.08	127.04 ± 18.90	131.29 ± 19.56	<0.001
Female	119.58 ± 17.50	122.85 ± 18.38	125.06 ± 19.95	128.76 ± 21.32	<0.001
Total	123.00 ± 19.24	123.97 ± 18.72	125.67 ± 19.65	129.45 ± 20.87	<0.001
**DBP (mmHg)**					
Male	79.52 ± 11.35	80.70 ± 11.10	82.22 ± 10.85	85.59 ± 10.58	<0.001
Female	75.13 ± 9.80	77.46 ± 9.65	79.23 ± 10.29	80.68 ± 10.97	<0.001
Total	77.86 ± 10.99	78.87 ± 10.43	80.14 ± 10.55	82.02 ± 11.08	<0.001
**FPG (mmol/L)**					
Male	5.10 ± 0.63	5.02 ± 0.58	5.14 ± 0.65	5.26 ± 0.67	<0.001
Female	5.18 ± 0.66	5.10 ± 0.61	5.13 ± 0.63	5.27 ± 0.65	<0.001
Total	5.12 ± 0.63	5.07 ± 0.60	5.13 ± 0.63	5.26 ± 0.65	<0.001
**HDL-C (mmol/L)**					
Male	1.54 ± 0.37	1.21 ± 0.23	1.09 ± 0.25	0.95 ± 0.27	<0.001
Female	1.65 ± 0.32	1.39 ± 0.26	1.22 ± 0.23	1.01 ± 0.20	<0.001
Total	1.58 ± 0.36	1.31 ± 0.26	1.18 ± 0.24	0.99 ± 0.23	<0.001
**TG (mmol/L)**					
Male	0.87 (0.73-1.03)	1.10(1.25-1.45)	1.77 (1.46-2.09)	2.83 (2.35-3.98)	<0.001
Female	0.74 (0.63-0.88)	0.93(1.05-1.19)	1.39 (1.23-1.58)	2.15 (1.81-2.76)	<0.001
Total	0.82 (0.69-0.99)	1.14(0.99-1.32)	1.47 (1.28-1.74)	2.35 (1.92-3.08)	<0.001
**WC (cm)**					
Male	72.76 ± 7.20	77.23 ± 8.26	81.11 ± 8.69	84.47 ± 8.52	<0.001
Female	69.40 ± 6.87	72.87 ± 7.85	76.57 ± 8.65	80.51 ± 8.80	<0.001
Total	71.50 ± 7.26	74.76 ± 8.31	77.95 ± 8.91	81.59 ± 8.90	<0.001
**BMI (kg/m**^**2**^**)**					
Male	21.15 ± 2.49	22.40 ± 2.84	23.47 ± 2.83	24.77 ± 3.06	<0.001
Female	21.61 ± 2.86	22.37 ± 3.08	23.38 ± 3.37	24.54 ± 3.17	<0.001
Total	21.32 ± 2.64	22.38 ± 2.98	23.41 ± 3.22	24.60 ± 3.14	<0.001
**WHtR**					
Male	0.44 ± 0.04	0.47 ± 0.05	0.49 ± 0.05	0.51 ± 0.05	<0.001
Female	0.45 ± 0.04	0.47 ± 0.05	0.49 ± 0.05	0.52 ± 0.06	<0.001
Total	0.44 ± 0.04	0.47 ± 0.05	0.49 ± 0.05	0.52 ± 0.06	<0.001

SBP, systolic blood pressure; DBP, diastolic blood pressure; FPG, fasting plasma glucose; HDL-C, high density lipoprotein cholesterol; TG, triglyceride; WC, waist circumference; BMI, body mass index; WHtR, waist-to-height ratio; VAI, visceral adipose index.

Median and inter quartile range for TG; Mean ± SD for SBP, DBP, FPG, HDL-C, WC, BMI, WHtR.

The incidence of diabetes among study population and diabetes risk with different body fatness indices are shown in Table 3. We found that the incidence of diabetes in the highest VAI group was 6.99%. As compared to individuals with the lowest VAI, those who had the highest VAI were at 2.55-fold risk of diabetes (95% CI: 1.58-4.11). We observed positive trends between diabetes risk and VAI level both for crude HR (HR = 1.38, 95% CI: 1.19-1.60) and adjusted HR (HRadj = 1.34, 95% CI: 1.15-1.56).

**Table 3:** The incidence and HR (95% CI) of diabetes risk with different body fatness indices groups

**Variables**	**New cases**	**Total subjects**	**Incidence (%)**	**HRcru (95% CI)**	HRadj* (95% CI)
**VAI**					
I	23	848	2.71	1 (referent)	1 (referent)
II	30	855	3.50	1.26 (0.73- 2.18)	1.22 (0.71-2.11)
III	45	871	5.17	1.86 (1.12-3.07)	1.75 (1.05-2.92)
IV	62	887	6.99	2.55 (1.58-4.11)	2.21 (1.35-3.61)
*HR* for trend	**(*p*** **< 0.001)**			1.38 (1.19-1.60)	1.34 (1.15-1.56)
**BMI (kg/m**^**2**^**)**					
Normal	93	2247	4.14	1 (referent)	1 (referent)
Overweight	54	985	5.48	1.30 (0.93-1.82)	1.15 (0.81-1.62)
Obesity	13	229	5.68	1.37 (0.76-2.44)	1.10 (0.60-2.00)
*HR* for trend	**(*p*** **= 0.102)**			1.22 (0.96-1.54)	1.07 (0.83-1.36)
**WC (cm)**					
Normal	110	2594	4.24	1 (referent)	1 (referent)
High	50	867	5.77	1.51 (1.09-2.10)	1.48 (1.04-2.10)
**WHtR**					
Normal	89	2224	4.00	1 (referent)	1 (referent)
High	71	1237	5.74	1.48 (1.08-2.02)	1.43 (1.03-1.97)

VAI, visceral adipose index; BMI, body mass index; WC, waist circumference; WHtR, waist-to-height ratio; HR, hazard ratio; CIs, confidence intervals.

*Adjusted for age, gender, SBP, DBP, smoking, alcohol drinking and DM-FH.

For BMI, WC and WHtR, significant association was observed between diabetes and WC (HRadj = 1.48, 95% CI: 1.04-2.10) and WHtR (HRadj = 1.43, 95% CI: 1.03-1.97); however, an insignificant relationship was observed for diabetes with BMI (HRadj = 1.07, 95% CI: 0.83-1.36).

The results of ROC analysis and AUC with its corresponding 95% CIs for VAI, WC, WHtR and BMI are shown in Table 4. The largest AUC was observed for VAI (AUC = 0.62, 95% CI: 0.58-0.66), following by WC (AUC = 0.55, 95% CI: 0.51-0.60), WHtR (AUC = 0.55, 95% CI: 0.50-0.59) and BMI (AUC = 0.54, 95% CI: 0.49-0.59).

**Table 4:** Comparison of AUC and corresponding 95% CI between VAI, WC, WHtR and BMI

**Variables**	**Standard error**	**AUC (95% CI)**	***p*-Value**
**VAI**	0.02	0.62 (0.58-0.66)	<0.001
**WC**	0.02	0.55 (0.51-0.60)	0.027
**WHtR**	0.02	0.55 (0.50-0.59)	0.046
**BMI**	0.02	0.54 (0.49-0.59)	0.072

AUC, area under curve; VAI, visceral adipose index; BMI, body mass index; WC, waist circumference; WHtR, waist-to-height ratio; CIs, confidence intervals.

## Discussion

In this prospective cohort study, we confirmed that obesity is an important risk factor of type 2 diabetes among a Chinese population, and found the increase of visceral adiposity was positively associated with metabolic disorders and diabetes risk with clear dose–response relationships. Compared to other body fatness indices, VAI was observed to be better in identifying the risk of diabetes than BMI, WC and WHtR.

The strong relationship between obesity and diabetes has been reported by many studies. Some researchers even use the term “diabesity” to describe their close associations [23]. Yang SL et al. found that people with large WC has 3.79-fold risk of diabetes than those whose WC were normal [24]. Yang WY et al. reported that the prevalence of diabetes in overweight group was 43% higher than that of normal people, and about 70% diabetes patients whose BMI were more than 25 kg/m^2^[3]. Clinical trials showed that even 5% weight loss was sufficient to prevent most obese subjects from impaired glucose tolerance and developing diabetes [25], especially for abdominal obesity.

The main harm caused by obesity is visceral adipose accumulation [26]. He et al. found that among men with normal WC, the incidence of MS in visceral obese people was significantly higher than that of normal group [27]. Fox et al. reported that both subcutaneous abdominal adipose tissue (SAT) and VAT were associated with increased odds ratio (OR) of MS. In women, the OR for VAT (OR = 4.7) was stronger than that for SAT (OR = 3.0); similar difference was shown for men (OR for VAT = 4.2; OR for SAT = 2.5) [8]. The molecular mechanism underlying this is unclear yet. It has been suggested that compared with subcutaneous fat, high visceral fat produces more free fatty acid, thus will increase the risk of IR and diabetes [8,28]. Fontana et al. reported that visceral adipose can secrete a large number of inflammatory cytokines, cells and adipokines, which may play important roles in the occurrence of IR and diabetes [26]. Liu et al. observed that visfatin, an adipokine which mainly produced by visceral adipose had the insulin-like effect, and has been proved to aggravate IR [29]. Moreover, as Masuzaki found in transgenic animals experiments, when 11 beta hydroxysteroid dehy-drogenase type 1(11βHSD-1) excessively expressed in fat cells, it would cause visceral adiposity and a series of metabolic disorders, which indicates that 11βHSD- 1 enzyme may have the same molecular basis with visceral obesity and metabolic disorders [30]. However, the mechanism between visceral adiposity and metabolic disorders still needs to be further elucidated.

Although visceral fat has been found independently associated with IR and diabetes, and could be used to estimate the risk of diabetes, it’s not easy to measure body VAT volumes. MRI and CT have been considered as the gold standard for VAT measurements, but it is obviously they are not suitable for large epidemiological studies because of the high cost and inconvenience. Different from direct VAT determination, VAI could be easily conducted in large-scale epidemiological studies and has been suggested as a useful surrogate marker of VAT. VAI includes physical (BMI and WC) and metabolic (TG and HDL-C) parameters, it may indirectly reflects other non-classical risk factors, i.e. altered production of adipokine, increased lipolytic activity and plasma-free fatty acids. Recently reported by Al-Daghri et al., VAI was negatively related with adiponectin value, this was the first report for the direct relations of VAI with adipose tissue secretion [31]. Some researches have proved that VAI could be used to predictive individual risk of IR, MS, acromegaly, cardiovascular disease (CVD) and diabetes [14,32-34]. Consistent with previous researches [14,31], our study also indicated that VAI is a useful surrogate marker to identify the risk of diabetes; individuals with high VAI were accompanied with increased risk of metabolic disorders and diabetes. The risk of getting diabetes at the highest VAI group was 2.55 folds higher as compared to the lowest VAI group.

According to the results of ROC analysis, the largest AUC was observed for VAI (AUC = 0.620, ***p*** < 0.001), following by WC, WHtR and BMI. Except VAI, AUC for other body fatness indices were all close to 0.5, which means the relatively lower predictive discriminatory power. Moreover, we observed positive trends between diabetes risk and VAI levels (HR = 1.38, 95% CI: 1.19-1.60), similar association was observed for WC (HRadj = 1.48, 95% CI: 1.04-2.10) and WHtR (HRadj = 1.43, 95% CI: 1.03-1.97), whereas only an insignificant adjusted HR was observed for BMI (HRadj = 1.07, 95% CI: 0.83-1.36). These findings suggested that VAI may be better in identifying diabetes risk. However, the ability of VAI in identifying diabetes risk was not found to be superior to WHtR in previous research which conducted among Tehran people [14]. This difference might be attributed to different study populations. It has reported that compared with Caucasians, Asians may have significantly higher risk of type 2 diabetes and CVDs despite substantially lower BMI [35]. Wang et al. found that Chinese men tend to have higher levels of FPG and DBP and significantly lower levels of HDL-C than Europeans [36]. Similar result was also found for Chinese women by Lear et al. [37]. In additional, several researches have proved that Chinese people may have higher VAT levels than European with a given body size [38,39]. Variations in lifestyle may also have effect on the relationship between VAT and diabetes risk among different populations besides hereditary factors [40]. To date, few published studies have explored relationship between VAI and diabetes risk and compared different body fatness indices with diabetes risk. Thus, the efficiency of VAI in predicting the risk of chronic diseases including diabetes still needs further verification from studies in different areas and different ethnicities.

Some limitations of our study need to be discussed. Firstly, because of the relative short time of follow-up and hence the relatively small number of new diabetes cases, the statistical power to examine the associations between VAI and diabetes may be insufficient. Secondly, IR was not measured in the present analysis therefore we were not able to directly examine the associations between VAI and IR. Thirdly, we did not examine 2-hour postprandial glucose, which may lead to the under diagnosis of some diabetic patients. Finally, residual confounders, an innate limitation of observational studies, could not be eliminated thus may increase the possibility that uncontrolled or inadequately measured confounders affected our results.

Despite these limitations, our study confirmed that obesity is an important risk factor of diabetes among Chinese population and found visceral adiposity is more important in disease occurrence. Compared to other indices for body fatness measurements, VAI is a good and convenience surrogate marker for visceral adipose measurement and could be used in identifying the risk of diabetes in large-scale epidemiologic studies.

## Abbreviations

AUC: Area under curve; BMI: Body mass index; CIs: Confidence intervals; CT: Computed tomography; CVD: Cardiovascular disease; DBP: Diastolic blood pressure; DM-FH: Family history of diabetes; FPG: Fasting plasma glucose; HDL-C: High density lipoprotein cholesterol; IR: Insulin resistance; MRI: Magnetic resonance imaging; MS: Metabolic syndrome; OR: Odds ratio; ROC: Receiver operating characteristic; HR: Hazard ratio; SAT: Subcutaneous abdominal adipose tissue; SBP: Systolic blood pressure; SD: Standard deviation; TG: Triglyceride; VAI: Visceral adiposity index; VAT: Visceral adipose tissue; WC: Waist circumference; WHtR: Waist-to-height ratio; 11βHSD-1: 11 beta hydroxysteroid dehy-drogenase type 1.

## Competing interests

The authors declare that they have no competing interests.

## Authors’ contributions

CC and YX contributed equally to analyses the data and draft the manuscript, participated in data management and cleaning. ZRG, JY, MW and XSH designed the study, directed its implementation and were responsible for data management and cleaning, quality assurance and control. MW and XSH reviewed and revised the manuscript critically. All authors read and approved the final manuscript.
